# Vitamin D deficiency is not related to eating habits in children with Autistic Spectrum Disorder

**DOI:** 10.3934/publichealth.2020061

**Published:** 2020-10-15

**Authors:** Maria Pia Riccio, Gennaro Catone, Rosamaria Siracusano, Luisa Occhiati, Pia Bernardo, Emilia Sarnataro, Giuseppina Corrado, Carmela Bravaccio

**Affiliations:** 1 Department of Translational Medical Science, Child and Adolescent Neuropsychiatry, University Federico II of Naples, Italy; 2 Department of Educational, Psychological and Communication Sciences. Suor Orsola Benincasa University, Italy; 3 Department of Translational Medical Science, Section of Pediatrics, University Federico II of Naples, Italy; 4 Department of Pediatric Neurosciences, Unit of Neuropsychiatry, Santobono-Pausilipon Children's Hospital, Naples, Italy

**Keywords:** Autism Spectrum Disorder, eating habits, nutritional status, 25 hydroxy vitamin D, vitamin D deficiency

## Abstract

**Background and aims:**

Autism Spectrum Disorder (ASD) is characterized by the impairment of communication and social interaction and by repetitive, restricted and stereotyped interests. ASD is often accompanied by comorbidities; eating disorders are frequent and imply important nutritional deficits (i.e. deficiencies of vitamins, minerals and fatty acids). Vitamin D has a critical role in neurodevelopment and serum levels in ASD are reported inadequate. A useful reference for setting up a correct diet in childhood is the food pyramid, which is inspired by the Mediterranean Diet (MD). The MD guarantees an intake of nutrients, considered optimal to maintain an adequate nutritional status. The aim of this study is to explore serum levels of Vitamin D and food habits (through MD adherence) in a sample of children with ASD and evaluate a possible correlation between these factors.

**Methods:**

study participants include 91 children 47 presenting ASD and 44 healthy typically-developing (TD) subjects, as control group. We evaluated serum level of Vitamin D in both group; anthropometric parameters (weight, height, body mass index—BMI—and growth percentile) and MD adherence have been explored, in order to investigate the correlation among those data and level of Vitamin D in children with ASD. Lastly, the association between Vitamin D levels and severity of ASD symptoms has been analysed.

**Results and conclusion:**

74% of ASD group presented blood levels of Vitamin D under 30 ng/ml (normal range 30–100 ng/ml). The analysis performed showed that the two groups were significant different regards Vitamin D levels (t = 2.24, p < 0.05), according to literature. 31.9% of children with ASD presented a condition of overweight and 12.6% a condition of obesity. Adherence to the MD was low in 25.5% of cases. No significant statistical correlation has been found between Vitamin D serum levels, anthropometric parameters and the adherence to MD in the ASD group.

## 1. Introduction

Autism Spectrum Disorder (ASD) is a developmental disorder which affects communication and behavior, characterized by impairment of communication and social interaction and by repetitive, restricted and stereotyped interests [1]. There are several comorbidities associated with ASD, both psychiatric and medical. Among the latter, a condition frequently associated are eating disorders, especially regarding food selectivity which may affect up to 70–80% of ASD children [2]. Food selectivity has been accurately defined by Bandini et al. [3], who reported that ASD children exhibit more food refusal and limited food repertoire than tipically-developing (TD) children. Moreover, in ASD food selectivity persists throughout childhood and the food repertoire remains narrowed [4]. Children with ASD eat a narrower range of food than TD children [5] and often they made food choices based on texture [6]. Proposed factors influencing food refusal are mainly related to the characteristics of food and they can be at least partially explained by bitter taste sensitivity [7].

Food refusal is often accompanied by important nutritional deficits that can also cause serious health problems. In detail, children with ASD frequently have deficiencies of vitamins, minerals and fatty acids. Using a prospective dietary data to study children with ASD, Edmond et al. observed that they eat less salad, fruits and in general less vegetables [8]. Bandini et al. found that children with ASD had inadequate intake of nutrients comparing to TD children with same age [3]. This poor supply can lead to lower concentrations of Vitamin A, Vitamin C, Folic Acid, biotin, calcium, zinc and iron in children with ASD [9]. In addition, serum concentration of pantothenic acid, vitamin B12, Vitamin E and Vitamin D have been found to be lower in ASD participants; in particular, levels of Vitamin D are significant lower compared to their peers [10]. Vitamin D is a fat-soluble vitamin existing in nature in the form of ergocalciferol (D2) and cholecalciferol (D3); it is converted to an active form in the liver and kidney. Vitamin D metabolites circulate in the blood linked to Vitamin D Binding Protein (DBP). Its action is mediated by the vitamin D receptor (VDR), a nuclear receptor expressed in over 30 different tissues. The recommended level of Vitamin D in both adults and children should be at least above 30 ng/ml [11]. The best available vitamin D sources in food are cod liver oil and fatty fish. In addition, mushrooms, fish, eggs, meat, and dairy products contain Vitamin D2 and D3 [12]. Vitamin D play a critical role in neurodevelopment, especially related to brain cell growth and differentiation and several works in literature have explored its role in the ASD etiology [13].

A wide variety of foods in the diet minimises the possible deficiency of a particular nutrient. The Mediterranean Diet (MD), considered as an Intangible Cultural Heritage of Humanity by United Nations Educational Scientific and Cultural Organization (UNESCO) in 2010, is a dietary pattern rich in plant foods, along with high to moderate intakes of fish and seafood, moderate consumption of eggs, poultry and dairy products, low consumption of red meat and a moderate intake of alcohol. MD ameliorates the intakes of micronutrients and is associated with better health effects.

The present study aims to explore serum levels of Vitamin D in children with ASD comparing to a control group of TD subjects. Nutritional status (auxological parameters) and eating habits (MD adherence), are also evaluated in order to investigate their relationship with Vitamin D levels in children with ASD. Lastly, we investigate the association between Vitamin D levels and ASD severity symptoms.

## 2. Methods

### 2.1. Subjects

Participants were a consecutive sample of children, along 12 months, referred to Department of Pediatrics—Unit of Child and Adolescent Neuropsychiatry, University Federico II of Naples, for an evaluation in a clinical suspicion of ASD. About 160 children received a full assessment, including historical informations (pregnancy, childbirth, birth weight, breastfeeding, weaning), structured clinical interviews and validated observations. Autism Diagnostic Observation Schedule-2 (ADOS-2) [14] was performed by a licensed clinician both to confirm diagnosis and to evaluate level of symptoms according to comparative score. To determine the development/intellective level, Griffiths Mental Development Scale (GMDS-ER) [15] or Leiter International Performance Test-Revised (Leiter-R) [16] were administered. To establish adaptive competence of all participants with ASD, parents were interviewed by Vineland Adaptive Behavior Scales—II edition (VABS-II) [17]. Diagnosis of ASD was formulated according to DSM-5th Edition [1], and through a period of 12 months, almost 100 children received a diagnosis of ASD.

The study was conducted in according to principles of Helsinki Declaration; ethical approval was obtained by the Ethics Committee of the University Federico II of Naples. Written informed consent was collected from parents or legal guardians of enrolled children for both clinical information collection and data acquisition and treatment.

Study participants included 91 children of which 47 had a diagnosis of ASD, whose parents signed informed consent form to participate to the study. Children were aged between 24 and 132 months (Mean age = 47.3 months; S.D. ± 15.38), and were both male (n = 33) and female (n = 14). According to DSM-5 criteria, clinical severity level was distributed as follows: 19.1% of cases were categorized as “Level 1” severity, that refers to clinical conditions in which a subject needs a support to have an adequate adaptation; 36.2% as “Level 2” severity, that refers to clinical conditions in which a subject needs a significant support to have an adequate adaptation; finally, 44.7% as “Level 3” severity, that refers to clinical conditions in which a subject needs a very significant support to have an adequate adaptation. According to the criteria of the DSM-5, other specifiers of the diagnosis were distributed as follows: 83% of cases presented ASD “With accompanying language impairment” (no intelligible speech, single words or isolated sentences); 61.7% presented ASD “With accompanying intellectual impairment” (according to results of developmental/intellectual/adaptive level, evaluated through GMSD-ER, Leiter-R and VABS-II). Detailed clinical features of ASD sample are described in Table 1. Inclusion criteria were a clinical diagnosis of ASD, age between 24 and 132 months; exclusion criteria included: advanced pubertal growth; epilepsy diagnosis or other neurological disorders; other chronic diseases (e.g. chronic intestinal diseases, malabsorption, etc.); major malformations or previous gastroenteric, urinary or respiratory surgery. 44 healthy TD subjects, aged between 24 and 132 months (Mean age = 65.23 months; S.D. ± 22.6) both males (n = 25) and females (n = 19), were recruited as control group, from those who attend daily minor surgery procedures (e.g. inguinal hernia, phimosis). Inclusion criteria were the absence of psychiatric diagnosis, age between 24 and 132 months. For the control group the same exclusion criteria were used.

**Table 1.** Clinical features of ASD Children.

	ASD (N = 47)
Male	33 (70.2) †
Age, months	47.3 (15.3) **
Autism Severity index (according DSM-5 severity levels)	
Low	9 (19.1) †
Mild	17 (36.2) †
Severe	21 (44.7) †
Verbal impairment (considering verbal as producing 5 or more worlds)	
Children without verbal impairment	8 (17) †
Children with verbal impairment	39 (83) †
Intellectual impairment (IQ/DQ < 70)	
Children without intellectual impairment	29 (61.7) †
Children with intellectual impairment	18 (38.3) †
ADOS-2, Mean Total score	18.5 (5.2) **
ADOS-2 (level of symptoms according comparative score)	
Low level	16 (34) †
Moderate level	21 (44.7) †
High level	10 (21.3) †

Note: ASD, Autism Spectrum Disorder; N., number; DSM-5, Diagnostic and Statistical Manual of Mental Disorders-5th Edition; IQ, Intellectual Quotient; DQ, Developmental Quotient; ADOS-2, Autism Diagnostic Observation Schedule-2. † Expressed as number of patients or controls and percentage in parenthesis; ** Expressed as Mean and Standard Deviation in parenthesis

All subjects were recruited from the same geographic area (Mediterranean coast of Campania region—Southern Italy), and were Caucasian from middle socioeconomic status.

### 2.2. Nutritional state assessment

Children were evaluated in a multidisciplinary setting (paediatrician, child psychiatrist, dietician). A dedicated module was created for collecting demographic and anamnestic data, current feeding informations (including those on special diets), clinical characteristics. Anthropometric parameters (weight, height, body mass index—BMI—and growth percentile) were collected; eating habits were evaluated through dietary diary (performed for 3 days) and adherence to the MD. The 3 days food diary consists in a collection of information regarding the type and quantity of food, drinks, condiments and supplements taken during the day (breakfast, lunch, dinner and snacks). Adherence to the MD was evaluated through KIDMED test (Mediterranean Diet Quality Index for children and adolescents) and KIDMED index was used to assess level of adherence [18]. The index ranges from 0 to 12 and is based on a 16-question test that can be self-administered or conducted by an interview. The sums of the values of the administered test are classified into three levels of score: (1) >8, optimal adherence to the MD (high adherence); (2) between 4–7, improvement necessary to adapt nutrition to the MD (moderate adherence); (3) ≤3, very low quality of adherence to the MD (low adherence).

Each patient was also investigated by blood samples, also for assessment of serum 25-hydroxyvitamin D [25(OH)D] concentration. Blood samples were collected in hospital for both ASD and TD children. Phlebotomy was conducted by a pediatric nurse, collection was made in fasting status, in the morning before breakfast. All samples were regularly sent for analisis to the university central laboratory, based on gold standard reference measurement procedures and certified reference materials. Serum levels of Vitamin D (tested through direct immunoassay by chemiluminescence) were classified as follow: deficient when <10 ng/ml; insufficient between 10–30 ng/ml; and normal between 30–100 ng/ml.

BMI was performed to classify children in four categories as follow [19]: underweight (< 5^th^ percentile), normal range (between 50^th^ and 85^th^ percentile), overweight (between 85^th^ and 95^th^ percentile) and obese (>95^th^ percentile). Aged within 24 and 36 months children were evaluated performing weight and height ratio and checking it on growth curve.

### 2.3. Statistical analysis

We applied The Statistical Package for Social Sciences software 25th edition (SPSS-25th ed.) to perform statistical analysis. Descriptive statistics (frequencies, percentages, mean and standard deviations) were used to describe the sample. An independent sample T-Test analysis was performed in order to compare auxological parameters and the level of Vitamin D between the ASD group and the control group. A chi-square test was used to compare categorical data (BMI categories, serum level of Vitamin D as categories). Bivariate correlational analysis (Spearman coefficient) was carried out to evaluate the relationship between severity of autistic symptoms, presence of intellectual impairment, verbal impairment and vitamin D values; adherence to the MD and vitamin D values.

## 3. Results

The groups consisted of 91 children, 47 ASD and 44 TD participants. No one was on special diet or took supplements on time of enrollment both in ASD children and in TD subjects.The main auxological and nutritional characteristics of the samples are reported in Table 2. Evaluation of BMI showed that 15/47 (31.9%) children with ASD presented a condition of overweight and 6/47 (12.8%) a condition of obesity. Only 2/47 (4.3%) children presented a condition of underweight. Whereas the control group, 4/44 (9.1%) subjects presented a condition of overweight, but there was none condition of obesity. Instead, 6/44 (13.6%) of TD children presented a condition of underweight. Comparing mean BMI value and frequencies distribution of BMI categories, the analysis revealed a significant difference between the two groups (p < 0.01). 29/47 (61.7%) children with ASD presented blood levels of Vitamin D indicative of a condition of insufficiency (insufficient level 10–30 ng/ml) and 6/47 (12.8%) presented blood levels of Vitamin D indicative of a condition of deficiency (deficient level <10 ng/ml). Just 12/47 (25.5%) subjects presented a level of vitamin D within normal range (normal range 30–100 ng/ml). Mean blood level of vitamin D in children with ASD was 22.7 ng/ml, indicative of a vitamin deficiency. In control group, 24/44 (54.5%) of TD children presented blood levels of Vitamin D indicative of a condition of insufficiency and 1/44 (2.3%) levels indicative of a condition of deficiency. 19/44 (43.2%) of TD subjects presented level of vitamin D in normal range. Mean blood level of vitamin D in TD children was 27.8 ng/ml. Comparing the mean of blood Vitamin D levels, there was a significant difference among the ASD and the TD subjects groups (t = 2.24, p < 0.05). The analysis revealed also a significant difference between age of the two groups. Therefore, a post hoc bootstrap analysis, stratified for the variable age, was performed in order to confirm the significant difference of vitamin D in the two groups. This analysis did not alter the previous result (t = 2.24, p < 0.01).

**Table 2.** Anthropometric parameters and Vitamin D level in ASD vs TD subjects groups.

	ASD (N = 47)	TD (N = 44)
Height(cm) **	104.6 (12.9)	111.3 (16.9)
Weight (Kg) **	18.5 (5.4)	19.9 (8.0)
BMI(Kg/m^2^) **	16.8 (1.7) *	15.7 (2.0)
Vitamin D(ng/ml) **	22.7 (12.4) *	27.8 (8.4)
BMI categories
Underweight (<5° percentile) †	2 (4.3) *	6 (13.6)
Normal Range (5–85° percentile) †	24 (51.1) *	34 (77.3)
Overweight (85–95° percentile) †	15 (31.9) *	4 (9.1)
Obese (>95° percentile) †	6 (12.8) *	0 (0.0)
Vitamin D level categories
Normal (30–100 ng/ml) †	12 (25.5)	19 (43.2)
Insufficient (10–30 ng/ml) †	29 (61.7)	24 (54.5)
Deficient (<10 ng/ml) †	6 (12.8)	1 (2.3)

Note: *p < 0.01; ASD, Autism Spectrum Disorder; TD, Typically-Developing; N., Number; BMI, Body Mass Index; † Expressed as number of patients or controls and percentage in parenthesis; ** Expressed as mean and Standard Deviation in parenthesis.

Food habits in children with ASD were detected through KIDMED test to explore adherence to a healthy diet, such as the MD. KIDMED index was used to evaluate adherence to the MD. 12/47 (25.5%) children presented a score ≤3 (low adherence to the MD), 31/44 (66%) presented a score between 4–7 (moderate adherence to the MD) and just 4/47 (8.5%) presented score >8 (high adherence to the MD) (Table 3).

To better characterize vitamin D deficiency in children with ASD, a correlation between vitamin D and KIDMED index was assessed but statistical analysis does not show a significant correlation. (Table 4).

Severity of clinical features was assessed according to DSM-5 (presence/absence of verbal impairment, presence/absence of intellectual impairment, level of symptoms according to ADOS-2 comparative score (as yet reported in Table 1). A correlation between the indicated variables and blood vitamin D levels in children with ASD was not statistically significant (Table 4).

**Table 3.** MD adherence index of ASD Children (N = 47).

KIDMED categories
Low adherence †	12 (25.5)
Moderate adherence †	31 (66)
High adherence †	4 (8.5)

Note: MD, Mediterranean Diet; ASD, Autism Spectrum Disorder; N., number; † Expressed as number of patients or controls and percentage in parenthesis.

**Table 4.** Correlation analysis between Vitamin D level, MD adherence and severity of clinical impairment in ASD.

	Adherence to mediterranean diet	Level of severity according DSM-5	Verbal impairment	Intellectual impairment	ADOS-2 comparative score
	Coeff.	Coeff.	Coeff.	Coeff.	Coeff.
Vitamin D	0.081	0.059	−0.127	0.102	0.118

Note: Table shows bivariate correlation analysis between mean serum vitamin D levels in children with ASD and adherence to MD, level of severity (according to DSM-5), verbal impairment, intellectual impairment, ADOS-2 comparative score. Results indicate that correlation between indicated variables and serum vitamin D levels is not statistically significant. Abbreviations: MD, Mediterranean Diet; ASD, Autism Spectrum Disorder; DSM-5, Diagnostic and Statistical Manual of Mental Disorders-5th Edition; ADOS-2, Autism Diagnostic Observation Schedule-2; Coeff., Spearman coefficient of correlation.

## 4. Discussion

Nutrition represents an important factor involved in maintaining an optimal state of health. A useful reference for setting up a correct diet in childhood is the food pyramid, which is inspired by the Mediterranean Diet [20]. The MD guarantees an intake of nutrients, optimal to maintain an adequate nutritional status. We focused our attention on eating habits of a sample of children with ASD, evaluating adherence to the MD, nutritional status, and a possible role of the MD adherence on vitamin D serum level. In the sample, an high percentage of ASD patients had an elevated BMI, which differed significantly from that in a TD subjects group. Moreover, adherence to the MD is low in children with ASD. Our results are in line with literature. In fact, several authors have confirmed that in ASD there is a higher consumption of high energy foods, greater preference for junk food, and lower consumption of healthy foods [21]. These eating habits, also linked to food selectivity, may lead to an increase in body weight up to a “state of obesity”. Therefore, poor feeding seems to be one of the possible reasons to explain the nutritional deficiencies in ASD.

The greater incidence of eating problems, particularly food selectivity in ASD, seems to be causedby many factors. For example, sensory difficulties can lead to atypical eating behaviours, whereas children may avoid certain foods due to the consistency and/or taste and eat only a limited variety [7]. Other data underlined the presence of gastrointestinal problems [22], food allergy and intolerance [23] in ASD, suggesting a possible role on feeding and on food choices in this population. However, there is no conclusive answer to this serious issue.

Our results show a significant difference between ASD and TD subjects regarding blood level of vitamin D. The ASD group present lower concentrations of vitamin D thanTD children. This data confirms what already reported in literature. Wang T et al. [24] performed a meta-analysis of clinical studies that explored the correlation between vitamin D levels and children with ASD. They reported a serum concentration of vitamin D significantly lower inchildren with ASD in comparison with healthy controls [10],[25]–[32].

This deficiency, together with other risk factors in children with ASD, such as reduced exercise and low exposure to the sun, restrictive diets, calcium deficiency, obesity and use of drugs, cause a low bone mineral density and a greater risk to develop metabolic syndrome and cardiovascular disease.

In our sample, low level of vitamin D did not relate with food intake and in particular with adherence to the MD. This data may suggest that the origin of Vitamin D deficiency is not related to food style, despite the inadequate feeding in ASD. Less UVB exposition can led to low synthesis of vitamin D, however children of our sample came from the same geographical area, on the Mediterranean coast, and were regularly exposed to sunlight. It should be taken into account that less endogenous production of vitamin D in ASD might depend by abnormal metabolism pathway possibly due to genetic polymorphisms [33]. Moreover other factors like digestion capacity, absorption, metabolic system, have a significant impact on nutritional status and so could influence vitamin D serum levels. In our samples, children did not present any chronic gastrointestinal disease or other conditions of malabsorption or malnutrition, as those have been considered exclusion criteria for enrollment. However,Vitamin D deficiency in ASD could be linked to insufficient intake derived yet in pregnancy [34], as reported in literature, and to a subsequent capability to replace deficiency during growth in early infant, thought food intake.

Gentile et al. [35] suggested a pathogenic model for ASD highlighting the role of an immune dysregulation as *primum movens* of this disease. Indeed, ASD may result from a complex interaction between genetic predisposition and environmental factors [36]. Among environmental factors, vitamin D may play a critical role because of his well-known effects on modulating immune response. In presence of these two factors (a genetic predisposition and vitamin D deficiency), a viral infection [35],[37] or a reactivation of the mothers can trigger an altered immune response that may lead to pathological neurodevelopment. Experimental evidences have been produced on this topic: gestational vitamin D deficiency has been related to an increase odd of ASD with Intellectual Disability (ID) offspring [38] and with a poorer motor and social development outcome [39]. Interestingly, higher neonatal Vitamin D levels were protective for ASD without ID, raises interesting questions about timing and critical windows to propose vitamin D supplementation [38].

Finally, no difference was observed in relation to the severity of symptoms inASD with or without deficiency of Vitamin D. This data disagrees from other literature results [25],[26],[28], reporting a positive correlation between vitamin D levels and clinical feature. Perhaps, the use of comparative score of ADOS-2 for the evaluation of level and gravity of ASD symptoms, such as the use of intellectual ability and verbal functioning, would indeed confer more effectiveness to the results that we obtained in comparison to other studies in literature which used less structured and reproducible evaluation tools.

Our results suggest that low adherence tothe MD may result in a difficulty compensating vitamin D deficiency, so that vitamin D supplementation has to be considered in addition to an balanced diet. It is very important to evaluate this nutritional parameter in regular clinical practice, not only for its posible role on neurodevelopment functions but also for the implications on the general state of health. In literature the experimental use of vitamin D is spreading, reporting an improvement the clinical features in children with ASD [40],[41]. In light of this is important to perform more studies on how the vitamin D may act in the etiopathogenesis and development of ASD and if it can become part of the complementary therapies, given the cost-benefit ratio and the therapeutic security.

Nonetheless some limitations catched our attention: a low sample size could have influenced the results observed, although they are in line with previous publications [29],[42]–[44]. ASD group and controls slightly differ for age, nonetheless this difference did not interfere with the results thanks to the bootstrap analysis, afterwards done to avoid statistical bias. Moreover, the data of the eating habits through the 3-day food diary were not presented. Future studies are needed in order to analyze these aspects and carry out a more accurate assessment even for the control group.

In conclusion, this study report that children with ASD present low adherence to the MD; this habit might imply overweight and obesity, that are observed in an higher percentage of ASD compared to TD children. Low serum level of vitamin D are confirmed in children with ASD; however, this data does not correlate to adherence to the MD in children with ASD.
